# Study on Dynamic Response Measurement of the Submarine Pipeline by Full-Term FBG Sensors

**DOI:** 10.1155/2014/808075

**Published:** 2014-05-26

**Authors:** Jinghai Zhou, Li Sun, Hongnan Li

**Affiliations:** ^1^School of Civil Engineering, Dalian University of Technology, Dalian 116024, China; ^2^School of Civil Engineering, Shenyang Jianzhu University, Shenyang 110168, China

## Abstract

The field of structural health monitoring is concerned with accurately and reliably assessing the integrity of a given structure to reduce ownership costs, increase operational lifetime, and improve safety. In structural health monitoring systems, fiber Bragg grating (FBG) is a promising measurement technology for its superior ability of explosion proof, immunity to electromagnetic interference, and high accuracy. This paper is a study on the dynamic characteristics of fiber Bragg grating (FBG) sensors applied to a submarine pipeline, as well as an experimental investigation on a laboratory model of the pipeline. The dynamic response of a submarine pipeline under seismic excitation is a coupled vibration of liquid and solid interaction. FBG sensors and strain gauges are used to monitor the dynamic response of a submarine pipeline model under a variety of dynamic loading conditions and the maximum working frequency of an FBG strain sensor is calculated according to its dynamic strain responses. Based on the theoretical and experimental results, it can be concluded that FBG sensor is superior to strain gauge and satisfies the demand of dynamic strain measurement.

## 1. Introduction

Submarine pipelines have been worldwide utilized in the field of offshore oil/gas exploitation [1] and have to endure long-term water load and accidental collisions. In addition, they are exposed to corrosion, erosion, and scour in harsh environment which all add to aggravate the damages.

More and more researchers have put great effort into the research of pipeline monitor in recent years and intelligent methods have been developed to monitor pipeline damage and positioning. Some network based on GPRS and methodology based on piezoelectric guided wave propagation were designed and used for pipeline monitoring [2–4]. However, the methods mentioned above have not been widely applied in engineering due to their shortcomings such as low sensitivity, poor long-term stability, and short lifespan.

Structural health monitoring (SHM) systems have shown great potential in monitoring the responses of a bridge system, diagnosing the current structural conditions, predicting the expected future performance, providing information for maintenance, and validating design hypotheses [5–7]. FBG sensor, which can be used as a part of structural health monitoring (SHM) systems, attracts more and more attention for its irreplaceable advantages such as simple structure, high sensitivity and accuracy, antielectromagnetic interference, strong reliability, good stability, quasidistributed measurement, easy network formability, and the capability available for real-time online monitoring [8, 9]. The application of optical fiber Bragg grating (FBG) sensors in structural health monitoring (SHM) of composite materials and structures has increased considerably in recent years [10].

Furthermore, submarine pipelines constructed in seismically active regions may be damaged under seismic load. The response of a submarine pipeline is a coupled vibration of liquid and solid interaction. The influencing factors for the dynamic responses of a submarine pipeline are very complex. The dynamic interactions may cause the structures to suffer from fatigue damages and/or catastrophic failures due to an excitation frequency approaching natural frequencies of the structure. Therefore, the evaluation of the dynamic responses of pipelines is extremely important in relation to safety and reliability.

It is necessary to understand the actual stress state of the pipeline in order to design submarine pipelines reasonably. Effective technical methods should be used to detect the signal under various loads and disease effects. Zeitoun et al. investigated the effects of applying the 2nd-order wave theory in predicting the kinematics on the pipeline dynamic response [11]. The dynamic responses of the pipeline under the 2nd-order wave theory and the linear wave theory were compared through finite elements analysis.

Recent research and development activities in structural health monitoring using FBG sensors have been critically reviewed [12]. The FBG sensor can well acclimatize itself to the severe environment surrounding the submarine pipeline due to its advantages. In monitoring structures in harsh environment, optical fiber sensors demonstrate superiority to conventional electric sensors in their immunity of electromagnetic interference [13].

Panopoulou et al. [14] used FBG sensors to detect the variations of the dynamic features of a structure made of two joint steel bars in the presence of artificial damage. A new system for structural health monitoring of composite aerospace structures based on real-time dynamic fiber Bragg gratings was developed by Capoluongo et al. [15]. Also, an intelligent health monitoring system of aerospace composite structures based on dynamic strain measurements was developed in order to identify the structural state condition in an exhaustive way [16]. The embedded FBG sensors applied as tools for modal analysis or in monitoring local and dynamic strains are demonstrated, which has paved the way for developing new methods in structural health monitoring [17].

Among the optical fiber sensors based on varied principles, fiber Bragg grating (FBG) sensors are the most promising candidates to effectively replace conventional strain gauges for long-term monitoring applications in harsh circumstances [18]. With the development of the natural gas industry and the establishment of network of gas pipelines, gas leakage has become a big problem in the field of security of gas pipeline network system. A new method based on FBG strain sensor has been proposed for leakage detection of natural gas pipeline [19–21]. Although FBG sensors have been applied to the monitoring of bridges, dams, piles, and so forth [22], there are few demonstrations in the continuous monitoring of the performance of submarine pipelines, and even fewer are the application of FBG accelerometers.

In this work, dynamic properties of FBG sensors are studied. Namely, the lag time and the maximum working frequency of FBG strain sensors are investigated by analyzing the transmission of the strain. Moreover, a model of the submarine pipeline is experimented to validate the reliability of FBG accelerometers and to test the dynamic properties of FBG strain sensors. Furthermore, the efficiency of frequency multiplexing techniques of FBG sensors for structural monitoring over long distance is demonstrated.

## 2. Dynamic Characteristics of FBG Strain Sensors

### 2.1. FBG Sensing Principle

A fiber Bragg grating is a periodic structure fabricated by exposing photosensitized fiber core to ultraviolet light. When the FBG is illuminated with a broadband light source, any induced strain in the FBG is encoded as a wavelength shift of the light reflected by the FBG. A resonance for light traveling down the FBG core satisfies the following Bragg equation:

(1) $$\begin{array}{c} \lambda_{\text{B}}=2n_{\text{eff}}\Lambda , \end{array}$$

where *λ*_B_ is the Bragg wavelength, or the wavelength of the light that is reflected by a fiber Bragg grating, *n*_eff_ is the effective refractive index of the fiber core, and Λ is the grating period. Both the effective refractive index and the grating period vary with changes in strain and temperature imposed on the fiber. The mechanism for applied strains to shift the Bragg wavelength is through expansion or contraction of the refractive index. These effects are well understood, and when adequately modeled, provide a means for predicting strain and temperature of the fiber. If only the dominant linear effects of these two factors on an FBG are considered, neglecting higher-order cross-sensitivities, the amount of Bragg wavelength shift can be represented by the linear relationship

(2) $$\begin{array}{c} \Delta \lambda_{\text{B}}=\alpha_{\varepsilon }\cdot \varepsilon +\alpha_{T}\cdot \Delta T, \end{array}$$

where *α*_*ε*_ is the strain sensitivity coefficient of the FBG sensor, *α*_*T*_ is temperature sensitivity coefficient, Δ*T* is the change of temperature, and *ε* is strain.

The strain coefficient *α*_*ε*_ of an FBG strain sensor packaged by a stainless steel tube is different from bare fibers since packaged FBG sensors suffer from strain loss when glued to host material with different Young's modulus. In our work, static loading tests were first performed to calibrate the strain coefficient of FBG sensors. The steel tube packaged sensor is glued onto the surface of a plexiglass plate with the epoxy resin, and then a tensile experiment was carried out. The plexiglass plate is loaded step by step from 0 *με* to 500 *με*. In the range of linear elasticity, the measured strains by the FBG strain sensor were considered consistent with those by bare FBGs and strain gauges. It was found that the coefficient of bare FBG on plexiglass plate is 1.02 × 10^−3^ and that of a tube-packaged FBG strain sensor is 6.789 × 10^−4^ [23].

### 2.2. Lag Time of Strain Wave Transmitting between Host Specimen and FBG Strain Sensors

Under tensile tests, strain wave transmits from host specimen to FBG strain sensor [24]. The whole propagation process consists of four steps: strain wave propagating in host specimen; strain wave propagating in adherence coating; strain wave propagating in FBG strain sensor.

(*1) Strain Wave Propagating in Host Specimen.* The propagation velocity of a strain wave in host specimen is

(3) $$\begin{array}{c} \nu =\sqrt{\frac{E}{\rho }}, \end{array}$$

where *ν* is the propagation velocity of a strain wave, *E* is the Young's modulus of measured elastic material, and *ρ* is the density of elastic material.

The propagation time *t* of a strain wave in the elastic material is

(4) $$\begin{array}{c} t=\frac{h}{\nu }, \end{array}$$

where *h* is the thickness of the elastic material.

(*2) Strain Wave Propagating in Adhesive Coating. *The time of a strain wave transmitting in adhesive coating to an FBG strain sensor is very short. Suppose the propagation velocity of a strain wave in adhesive layer (epoxy resin) is 1000 m/s and the thickness of adhesive layer is 0.03 mm; thus the propagation time in the adhesive layer is

(5) $$\begin{array}{c} t_{\text{adhesive}}=\frac{h}{\nu }=\frac{0.03}{1000\times 1000}=3\times 10^{-8}\;\text{s}. \end{array}$$

(*3) Strain Wave Propagating in an FBG Strain Sensor. *The outer, middle, and interior layers of the packaged FBG strain sensor are steel tube, epoxy resin, and a bare FBG, respectively. The wave propagates via the tube and epoxy resin and then reaches the glass fiber (bare FBG). Assuming that the radius of the steel tube *R* is 0.5 mm, the thickness of the tube m is 0.15 mm, the diameter of the bare FBG is 0.25 mm, *ν* = 4500 m/s in steel, and *ν* = 1000 m/s in epoxy resin, consequently, the propagation time of a strain wave in the steel tube is

(6) ttube=hν=m4500×1000+R−m−d1000×1000=0.154500×1000+0.5−0.15−0.251000×1000=1.33×10−7 s.

The lag time of the tube-packaged FBG strain sensor is

(7) tt=tadhesive+ttube=3×10−8+1.33×10−7=1.63×10−7 s.

It can be seen that the lag time of strain propagation is very short between elastic material and bare FBG, which can be ignored in engineering.

### 2.3. Maximum Working Frequency of an FBG Strain Sensor

**Figure 1:**
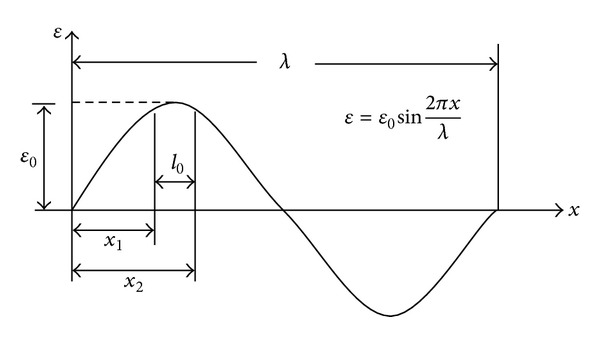
The response property of FBG strain sensor to sine strain wave.

To obtain the maximum working frequency of FBG strain sensor, sine strain wave and step strain wave are assumed, respectively.

(*1) Sine Function of Strain Wave. *The FBG strain sensor response property to a sine strain wave is shown in Figure 5. The strain measured by the FBG sensor is the average of strains along total effective length. Therefore, the amplitude of the measured strain is less than peak value of the transmitted actual strain wave. In Figure 1, the maximum value of the measured strain can be obtained, only when the FBG strain sensor gets across the middle position of the strain wave with the highest amplitude. With the strain wavelength *λ* and packaged length *l*_0_, the coordinates of sensor ends are *x*_1_ = (*λ*/4)−(*l*_0_/2) and *x*_2_ = (*λ*/4)+(*l*_0_/2), respectively. So *ε*_*p*_ as the average measured strain of effective length is the maximum value; namely,

(8) εp=∫x1x2ε0sin(2π/λ)x dxx2−x1=−λε02πl0(cos⁡2πλx2−cos⁡2πλx1)=λε0πl0sinπl0λ.

*e* is defined as the error of measured strain and actual strain

(9) $$\begin{array}{c} e=\left|\frac{\varepsilon_{p}-\varepsilon_{0}}{\varepsilon_{0}}\right|=\left|\frac{\lambda }{\pi l_{0}}\sin\frac{\pi l_{0}}{\lambda }-1\right|. \end{array}$$

Obviously, the error between measured and actual strains increases with the increase of the length of sensors.

For measured elasticity structure,

(10) $$\begin{array}{c} \lambda =\frac{\nu }{f}, \end{array}$$

where *ν* is the wave velocity of strain sensor and *f* represents the vibration frequency of a measured structure and that is the maximum working frequency of FBG strain sensor.

For FBG strain sensor,

(11) $$\begin{array}{c} \lambda =nl_{0}, \end{array}$$

where *n* is the ratio between strain wavelength and the length of FBG strain sensor (*n* = *λ*/*l*_0_).

Substitution of (11) into (10) yields the following equation:

(12) $$\begin{array}{c} f=\frac{\nu }{\left(nl_{0}\right)}. \end{array}$$

**Figure 2:**
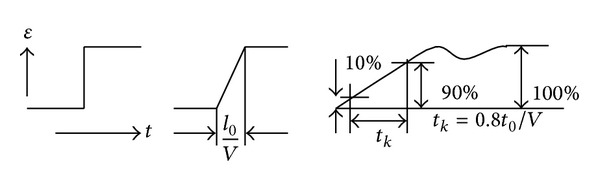
The response property of FBG strain sensor to step wave.

Note that in (12), this equation simply shows that the working frequency of an FBG strain sensor relates to *ν* and *n*.  Table 1 lists the maximum frequencies of an FBG strain sensor in different materials (*n* = 20). 

(*2) Step Function of Strain Wave. *Suppose the strain wave is a step wave, and Figure 2 presents the response property of an FBG strain sensor to a step strain wave.

There is a lag time for the measured strain to reach the highest amplitude because of the travelling time for the strain wave across the whole length of the FBG sensor. The theoretical output wave and real output wave are shown in Figure 2. *t*_*k*_ is the rising time (which is the time of the output measured value changing from 5 percent or 10 percent of the final stable value to 95 percent or 90 percent of the final stable value). Suppose that *t*_*k*_ is the time of the output value changing from 10 percent to 90 percent of the final stable value, *t*_*k*_ = 0.8*l*_0_/*ν*. The working frequency of the FBG sensor is *f* = 0.35/*t*_*k*_ for the step wave input from Sun et al.; that is,

(13) $$\begin{array}{c} f=\frac{0.35\nu }{0.8l_{0}}=0.44\frac{\nu }{l_{0}}. \end{array}$$

From (13), it can be seen that the working frequency of an FBG strain sensor relates to *ν* and *l*_0_.  Table 2 lists the maximum frequencies of an FBG strain sensor in different materials.

(*3) In Case That the Strain Wave Is in Other General Forms.* For other general wave forms, the minimum value of the results from computing (12) and (13) is supposed as the working frequency of an FBG strain sensor. Function *f*(*x*), in which the periodic time is *T*, can be expanded into a trigonometric series as follows:

(14) $$\begin{array}{c} f=\frac{a_{0}}{2}+\overset{\infty }{\underset{n=1}{\sum }}\left(a_{n}\cos n\omega x+b_{n}\sin n\omega x\right), \end{array}$$

where *ω* = 2*π*/*T*, *a*_0_ is constant, and

(15) $$\begin{array}{c} a_{n}=\frac{2}{T}\overset{T/2}{\underset{-T/2}{\int }}f\left(x\right)\cdot \cos n\omega x\;dx,\;\begin{array}{cc} n=0,1,2,\ldots ; & \end{array}\\ b_{n}=\frac{2}{T}\overset{T/2}{\underset{-T/2}{\int }}f\left(x\right)\cdot \sin n\omega x\;dx,\;n=0,1,2,\ldots . \end{array}$$

Consequently, the error analysis of measured and actual strains *e* can be calculated in a similar manner like that of a sine wave ((8) and (9)).

## 3. Submarine Pipeline Model and FBG Sensor System

### 3.1. The Physical Model

**Figure 3:**
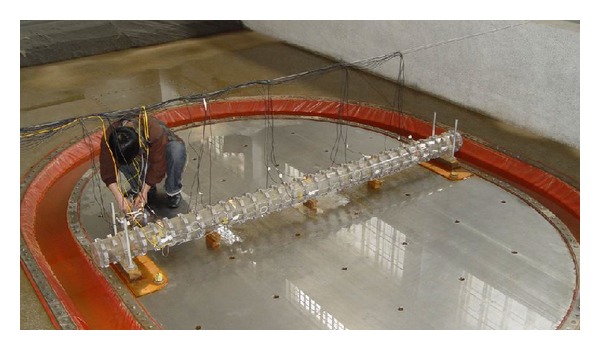
Underwater shaking table and submarine pipeline model in experiment.

**Figure 4:**
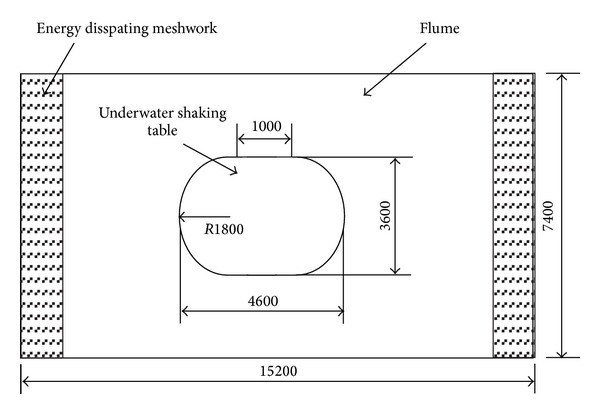
The sketch of the underwater shaking table (units: mm).

The physical model of a submarine pipeline is fabricated of plexiglass tubes as shown in Figure 3. The outer diameter of the model is 110 mm, the thickness of the tube is 2.8 mm, and Young's modulus is 5 GPa.  Figure 4 is a sketch of the underwater shaking table.

### 3.2. Underwater Shaking Table

Vibration experiments of the submarine pipeline model were performed on the underwater shaking table [25]. The movable part of the shaking table lies in the middle of a flume in which maximum water depth allowed is 1.0 m. The meshwork of the energy dissipation is installed in each side of the flume along horizontally excited direction in order to avoid the influence of water wave reflection.

### 3.3. Types of FBG Sensors and the Monitoring System

**Figure 5:**
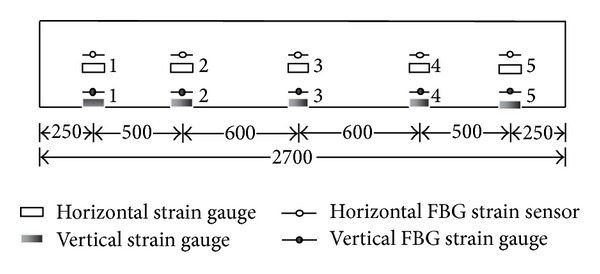
Position of strain sensors (units: mm).

**Figure 6:**
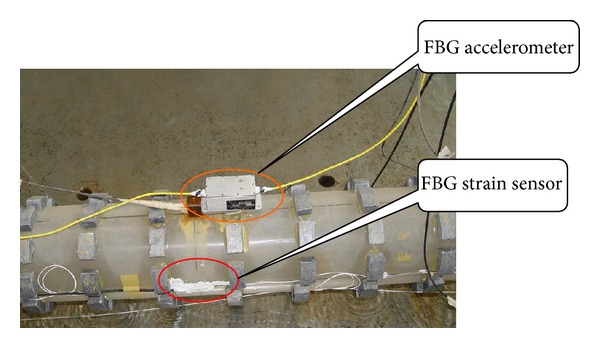
Photo of accelerometer on submarine pipeline model.

Three different types of FBG sensors, for measuring temperature, strain, and acceleration, respectively, were used in the experiments to assess their performance in dynamic measurements under various measurement tasks. The FBG stain sensors can be divided into two types: one is bare FBG sensor and the other is tube-packaged FBG sensor. There were totally ten FBG strain sensors used in our experiments, among of which five were bare FBG sensors and the other five were tube-packaged FBG sensors. Figure 5 shows the positions of strain sensors on the pipeline model. Moreover, two FBG accelerometers were used, one of which was mounted on the surface of the shaking table and the other is placed at the top of the model (shown as Figure 6). A separate FBG temperature sensor, which was laid on the ground freely, was introduced mainly for the compensation of FBG wavelength shifts due to variation of environmental temperature.

### 3.4. Remote Monitoring

To test the potential feasibility of FBG sensors for long distance structural health monitoring, a demodulation unit (FBG-SLI) for FBG sensors and a laptop PC were set up in an office, which locates around one hundred meters away from the experiment model. The optical signals from the thirteen FBG sensors were multiplexed in four multiplexing fiber units, and transmitted through single mode optical fibers to the demodulation unit in the relative remote office. The decrease of intensity of light over one hundred meters in our experiments is almost invisible and can be surely neglected. Such observations show that FBG sensors are suitable for remote structural health monitoring when the transmitting single mode optical fibers are bitterly curved.

## 4. Dynamic Loading Tests

Various tests of the submarine pipeline model on the shaking table in our laboratory were conducted to validate its performance. At first, swept sine excitations were employed to identify the first several natural frequencies of the submarine pipeline model.

### 4.1. Strain Response

The length of an FBG strain sensor *l*_0_ in this experiment is 40 mm; *ν* is 1700 m/s in plexiglass. Let *n* = 20. From (12), the highest working frequency is *f* = *ν*/(*nl*_0_) = 1700/(20 × 0.4) = 2.125 kHz. From (13), the highest working frequency is *f* = 0.44*ν*/*l*_0_ = 18.7 kHz; hence, in this experiment, the highest working frequency of an FBG sensor is *f* = 2.125 kHz, and the maximum vibration frequency of the shake table is 50 Hz; the FBG strain sensor can sufficiently measure the dynamic strains of the model.

**Figure 7:**
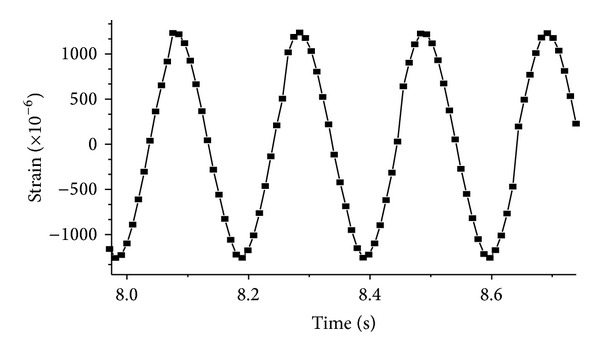
Strain induced by sine wave.

**Figure 8:**
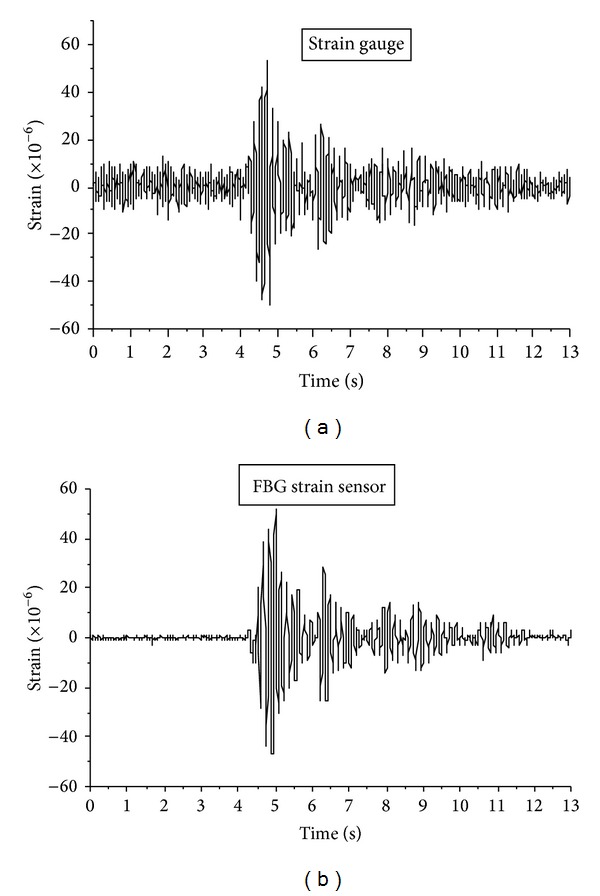
Strain course induced by El Centro wave.

**Figure 9:**
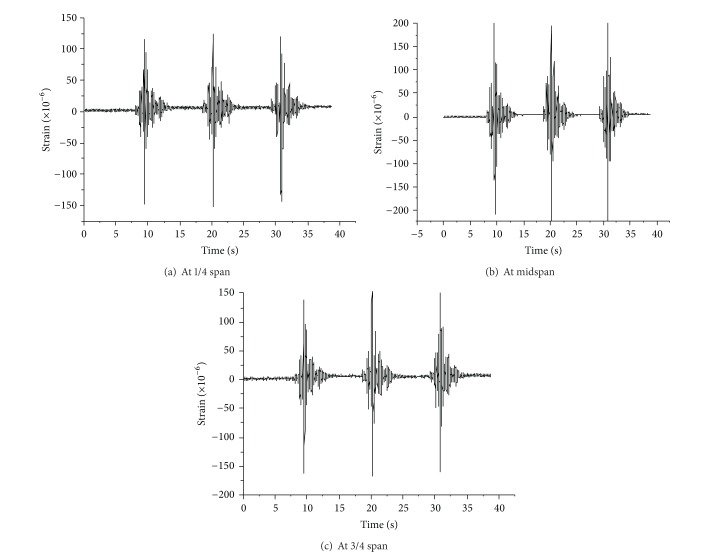
Strain course induced by Northridge wave.

Figure 7 shows the horizontal strain at midspan measured by an FGB strain sensor. The dynamic input was a sine wave in horizontal direction with the frequency of 4.8 Hz. It can be seen from Figure 7 that the tendency of the strain response is also a sine wave.  Figure 8 is the strain history induced by the El Centro wave. It can be seen that the horizontal strain response measured by FBG sensor is the same as the one monitored by electric strain gauge at the midspan. For the electromagnetic interference of environment, the noise of electric strain gauges is about 25 *με*. It is much larger than the noise of an FBG sensor, which is only 2 *με*. Compared with electric strain gauges, an FBG sensor shows its special advantage of immunity to electromagnetic interference and good capability in measuring the dynamic strain of vibration system with low frequencies. The dynamic excitation in Figure 9 is the Northridge wave in horizontal direction, and the strain at 1/4 span is symmetrical with that at 3/4 span.

### 4.2. Acceleration Response

**Figure 10:**
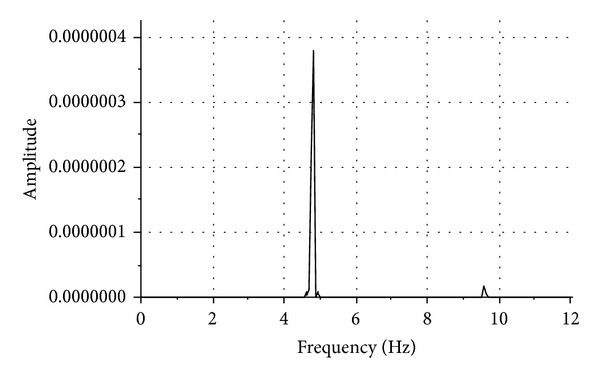
Power density spectrum of the acceleration response of the shaking table for sine excitation.

**Figure 11:**
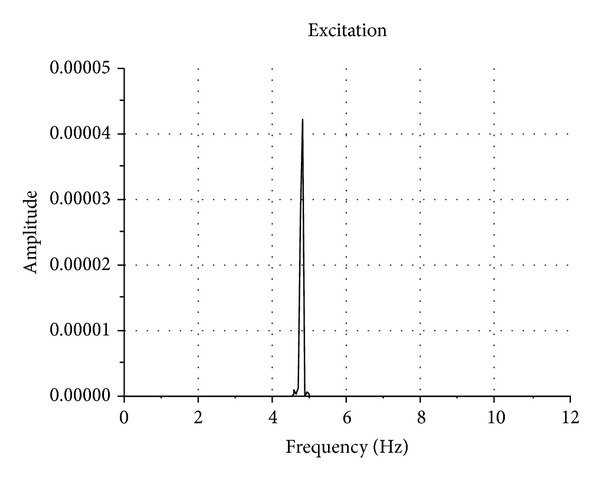
Power density spectrum of the acceleration response of the model for sine excitation.

Figures 10 and 11 show the power spectrum density functions of the FBG acceleration responses on the surface of the shaking table and on the top of the submarine pipeline model, respectively. The submarine pipeline model is excited by the shaking table with sine waves of 4.8 Hz underneath. From these figures, we can see that the resonant frequency is round 4.8 Hz, which approximately is equal to the excitation frequency.

However, there is a problem associated with the demodulation unit currently we are using. That is, the higher sampling frequency was fluctuating due to the hysterics of the PZT for its relative small linear range. Resolution and accuracy had to be sacrificed for the sake of the stableness of sampling time intervals, especially for the FBG accelerometers.

## 5. Conclusions and Discussions

This paper computes the lag time and the maximum working frequency of FBG strain sensors applied to submarine pipeline monitoring. Based on the analysis, the maximum working frequency of an FBG strain sensor satisfies the requirement of dynamic strain measurements. In other dynamic strain measurements, the working frequency of FBG strain sensor can be obtained by using (12) and (13).

Dynamic loading tests were performed in this paper to validate the feasibility of using packaged FBG as reusable strain sensors and accelerometers. Underwater seismic shaking table was utilized to provide the appropriate excitations. Preliminary results show that the FBG sensors mounted on the surface of the host material to be measured have advantage over the classical strain gauges with respect to accuracy and anti-interferences besides its superior ability for long distance structural health monitoring.

## Figures and Tables

**Table 1:** Maximum working frequency of FBG strain sensor (strain wave is sine wave).

λ/*l*_0_ (*n*)	20			
Effective length *l*_0_ (mm)	20	25	30	40
Sensor is glued onto steel *f* (kHz) (ν = 4500 m/s)	11.25	9	7.5	5.625
Sensor is glued onto plexiglass *f* (kHz) (ν = 1700 m/s)	4.25	3.4	2.83	2.125
Sensor is glued onto concrete *f* (kHz) (ν = 3500 m/s)	8.75	7	5.83	4.375
Sensor is glued onto rubber *f* (kHz) (ν = 30 m/s)	0.075	0.06	0.05	0.0375

**Table 2:** The maximum working frequency of FBG strain sensor (strain wave is step wave).

Effective length *l*_0_ (mm)	20	25	30	40
Sensor is glued onto steel *f* (kHz) (ν = 4500 m/s)	99	79.2	66	49.5
Sensor is glued onto plexiglass *f* (kHz) (ν = 1700 m/s)	37.4	29.92	24.93	18.7
Sensor is glued onto concrete *f* (kHz) (ν = 3500 m/s)	77	61.6	51.33	38.5
Sensor is glued onto rubber *f* (kHz) (ν = 30 m/s)	0.66	0.528	0.44	0.33
